# A Triad of Shoulder Injuries Following Cardioversion: A Case Report

**DOI:** 10.7759/cureus.52704

**Published:** 2024-01-22

**Authors:** Eng Kee Tan, Khairil Anwar Ahmad Hanif, Siti Munira Seri Masran, Fahrudin Che-Hamzah

**Affiliations:** 1 Orthopaedics, Ministry of Health Malaysia, Serdang, MYS; 2 Orthopedics and Traumatology, Hospital Pengajar Universiti Putra Malaysia, Serdang, MYS

**Keywords:** bankart lesion, supraspinatus tear, shoulder dislocation, case report, cardioversion

## Abstract

Transthoracic defibrillation and cardioversion are commonly used techniques to resuscitate a patient during acute cardiac arrhythmic events. There are numerous complications associated with these procedures. We report a previously unreported complication where a patient suffered from a supraspinatus tear after cardioversion for ventricular tachycardia. There are numerous complications associated with these procedures. We report a previously unreported complication where a middle-aged Chinese patient with no previous trauma history suffered from a supraspinatus tear after cardioversion for ventricular tachycardia.

## Introduction

Transthoracic defibrillation and cardioversion are commonly performed during acute cardiac arrhythmias. Energy and current requirements depend on the arrhythmia type. For ventricular tachycardia (VT), the recommended level ranges between 70 and 200 Joules [1].

Cardioversion is associated with various complications. These include cardiac arrhythmias, pulmonary edema, and burns. During cardioversion, the electrical stimulation may cause skeletal muscle contraction. If these contractions are too vigorous, they can cause musculoskeletal injuries.

This case report highlights a triad of shoulder injuries consisting of anterior shoulder dislocation (humerus head displaced anteriorly relative to glenoid fossa of scapula), Bankart lesion (injury to the anteroinferior aspect of glenoid labrum), and partial supraspinatus tendon tear as another possible complication of cardioversion. Such a phenomenon is previously unreported.

## Case presentation

The patient is a 45-year-old Chinese male who initially presented with an acute onset left-sided chest pain. He had no known underlying illness prior to presentation and had no preceding trauma or injury. He was diagnosed with a Killip IV acute inferior myocardial infarction and was thrombolyzed with streptokinase. He then developed VT and underwent cardioversion.

**Figure 1:**
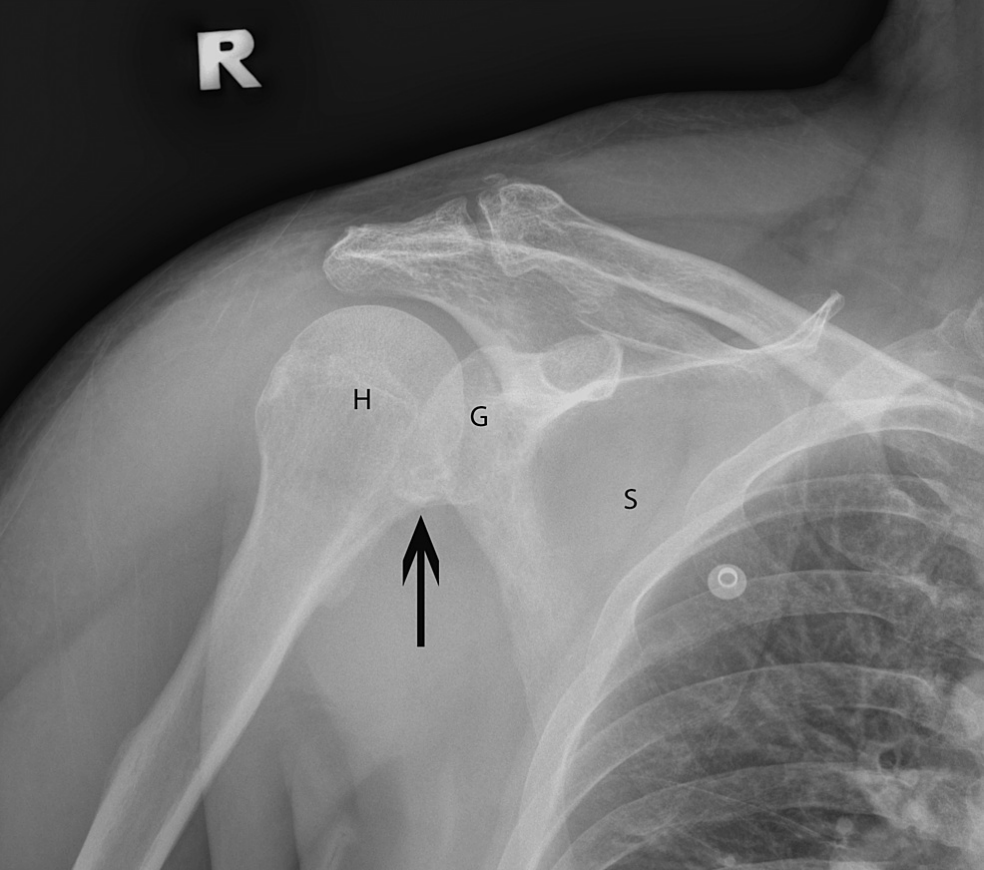
Plain radiograph of the right-shoulder anterior-posterior view demonstrating a Bankart lesion over the glenoid fossa of the right scapula. H: Humerus head; G: Glenoid fossa; S: Scapula; Black arrow: Bankart lesion

Upon resolution of the initial acute event, the patient complained of right shoulder pain, which was absent prior to admission. It was associated with weakness of the affected shoulder and swelling over the superior-lateral aspect, just distal to the acromion process. Abduction of the shoulder was limited with tenderness over the supraspinatus insertion area and a positive Dugas test. Plain radiographs revealed a Bankart lesion over the right glenoid (Figure 1).

**Figure 2:**
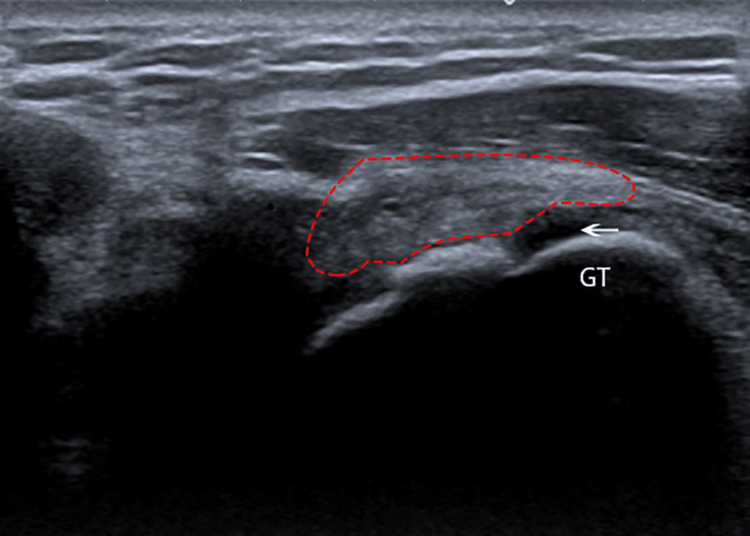
Ultrasound of the shoulder revealing a partial tear of the supraspinatus tendon from the greater tubercle of the humeral head. GT: Greater tubercle of humerus; Red outline: Supraspinatus tendon; White arrow: Partial tear of supraspinatus tendon from its attachment

An ultrasound of the right shoulder revealed a partial tear of the supraspinatus tendon with a long head of biceps tendonitis (Figure 2). He was placed on an armsling for his right shoulder and recovered well during follow-up after physiotherapy without the need for surgical intervention.

## Discussion

The shoulder joint is a ball and socket joint articulating the scapula and proximal humerus. It relies on multiple stabilizers, one of which is the rotator cuff that comprises four muscles - supraspinatus, infraspinatus, teres minor, and subscapularis [2].

Rotator cuff injuries tend to be disabling for the patient, as they lead to shoulder dysfunction due to secondary weakness caused by pain. Supraspinatus tears are the most common form of rotator cuff injuries and commonly result from a traumatic incident or a sports-related injury, and they are a well-known complication of shoulder dislocations [3].

Anterior shoulder dislocations are also commonly associated with Bankart lesions. Widjaja et al. reported an incidence of up to 85% in their study. They concluded that Bankart lesions are more likely to occur in anterior shoulder dislocations [4].

Transcutaneous cardioversion, though life-saving, can be associated with numerous complications. The more commonly encountered adverse events include transient ventricular dysfunction, arrhythmia, pulmonary edema, thromboembolism, and skin complications, such as erythema, burns, and pain [5].

Uncommonly, shoulder injuries can result as a complication of cardiopulmonary resuscitation, cardioversion, and seizure-like disorders. Gosens et al. reported two incidents of posterior shoulder dislocations following epileptic seizures. They stated that these injuries can be caused by uncontrolled muscular contractions, such as seizures or electric shock therapy [6]. In cardioversion, the electrical current involved in the usage of the device can potentially cause increased levels of muscular contractions, leading to potential musculoskeletal injuries.

Ould-Ahmed et al. reported one such incident of shoulder dislocation after cardioversion. The patient in this report had received cardioversion under propofol-induced general anesthesia for atrial fibrillation. It is likely that the vigorous muscle contractions from cardioversion caused the shoulder dislocation when it occurred in a generalized hypotonic state caused by anesthesia [7].

This complication is not limited to external cardioverters and can occur in subcutaneous implantable cardioverter-defibrillator devices (S-ICD). Two incidents of shoulder dislocation were reported after S-ICD testing. The electrical current generated by the S-ICD causes increased pectoralis major contraction, leading to adduction and internal rotation of the humerus, resulting in an increased risk of anterior shoulder dislocation [8].

Kam et al. also reported on a patient who sustained scapula and proximal humerus fractures after cardiopulmonary resuscitation and cardioversion. There was no direct trauma to the affected region during CPR, and it was postulated that the fractures occurred due to the cardioversion causing sustained muscle contraction of the shoulder girdle muscles [9].

Our case demonstrated an occurrence of an anterior shoulder dislocation, which was associated with a Bankart lesion and partial supraspinatus tendon tear. The dislocation was postulated to occur during the cardioversion. Although the dislocation had reduced spontaneously, the presence of a Bankart lesion and partial supraspinatus tear supports the diagnosis of an anterior shoulder dislocation. The triad of shoulder injuries sustained by the patient following cardioversion had never been reported before.

## Conclusions

In conclusion, cardioversion from an acute cardiac event can be associated with numerous complications. In this case, we reported a triad of injuries to the shoulder following transthoracic defibrillation. The triad consists of an anterior shoulder dislocation, a Bankart lesion, and a partial supraspinatus tendon tear. These injuries had never been reported before following cardioversion.
